# Correlation Study between Levels of Gastrin, Serum IGF-1, and GHBP and Growth and Development in Children with Short Stature Based on Big Data Analysis

**DOI:** 10.1155/2022/4614099

**Published:** 2022-08-25

**Authors:** Chen Hua, Dan Yu

**Affiliations:** ^1^Department of Child Healthcare, Yantai Mountain Hospital, Yantai, 264000 Shandong, China; ^2^Department of Pediatric General Surgery, Qingdao Women and Children's Hospital, Qingdao, 266034 Shandong, China

## Abstract

**Objective:**

To analyze the correlation between the levels of gastrin, serum IGF-1, and GHBP and growth and development in children with short stature (SS) using the big data.

**Methods:**

By means of retrospective analysis, the clinical data of 42 children with SS admitted to our hospital from October 2020 to October 2021 were selected as the study group, while 30 children with the healthy physical examination results in the corresponding period were selected as the control group to measure the growth and development indices and the levels of gastrin, serum IGF-1, and GHBP. The Pearson correlation analysis was used for the relationship between the levels of gastrin, serum IGF-1, and GHBP and growth and development indices in children with SS, and the targeted intervention measures were formulated by the analysis of experimental data.

**Results:**

Compared with the study group, the height, weight, and bone mineral density (BMD) *Z*-scores of children in the control group were obviously higher (*P* < 0.001). The levels of gastrin, serum IGF-1, and GHBP in the study group were markedly lower than those in the control group (*P* < 0.05). The Pearson correlation analysis showed that the gastrin, serum IGF-1, and GHBP of children were positively correlated with growth and development indices (*P* < 0.001). The levels of gastrin, serum IGF-1, and GHBP in children were distinctly improved after treatment (*P* < 0.05).

**Conclusion:**

The gastrin, serum IGF-1, and GHBP are closely related to the SS, and the effective clinical intervention can better improve the above indicators of children to promote their growth and development.

## 1. Introduction

Short stature (SS), a more common disease in children, refers to a child whose height is 2 standard deviations below the average height for the same age, gender, and race or below the 3rd percentile of normal children growth curve [1]. The specific etiology of children with SS that have no potential pathological condition is not clear, speculating that SS is a multigenic disease, and it is less likely to reach normal height after adulthood without timely treatment. The occurrence of SS will seriously affect the physiological, psychological, and intellectual abilities of children and bring heavy psychological and economic burden to their families, so that the active exploration of its pathogenesis can benefit these children. The disorder of skeletal development is one of the main causes of SS, and hereditary metabolic diseases, malnutrition, and other chronic diseases are also the risk factors for SS [2]. Insulin-like growth factor-1 (IGF-1) and growth hormone binding protein (GHBP) are closely related to the growth and development of young children. Among them, IGF-1 as a kind of peptide for promoting cells growth is similar to structure and function of insulin, which has an important promotion in cell proliferation and differentiation and individual growth and development [3, 4]. GHBP is formed by the decomposition of extracellular components of growth hormone receptor (GHR) by proteolytic enzymes, which plays an important regulatory role in human growth [5]. Gastrin is a newly discovered natural endogenous ligand for the growth hormone secretagogue receptor (GHS-R), which acts on pituitary and hypothalamus. It not only has an endocrine function but also promotes the appetite and enhances the gastrointestinal motility [6, 7]. Big data analysis is a method that predicts the possibility of events by comparing and analyzing the data obtained from the experiments and studies the correlation between data and data, with the instructive and representative analysis results [8]. At present, there are few studies on gastrin, serum IGF-1, and GHBP in children with SS. This study analyzed the correlation between the above indexes and the growth and development in children with SS by the big data, in order to provide a reference for the clinical diagnosis and treatment of the disease. The reports were as follows.

## 2. Materials and Methods

### 2.1. General Information

42 children with SS admitted to our hospital from October 2020 to October 2021 were selected as the study group with the age range of 2-13 years old and the average age of (7.02 ± 3.32) years old, including 23 males and 19 females. At the same time, 30 children with the healthy physical examination results in the corresponding period were selected as the control group with the age range of 2-13 years old and the average age of (7.13 ± 3.84) years old, including 17 males and 13 females. The study was approved by the hospital ethics committee and in line with the Declaration of Helsinki (2013) [9].

### 2.2. Inclusion and Exclusion Criteria

Inclusion criteria. (1) Children were in accordance with the relevant diagnostic criteria of SS, with the age less than 13 years old. (2) Children had the symmetrical stature and no chronic organic disease. (3) Children had the normal intelligence development.

Exclusion criteria. (1) Children with the liver, kidney and intestinal diseases; (2) children with the abnormal thyroid function; and (3) children with the tumor, mental disorders, and obesity.

### 2.3. Methods

#### 2.3.1. Detection of Laboratory Indexes

The fasting venous blood of children (6 ml) was collected from 8 : 00 to 10 : 00 in the morning and placed in three tubes without anticoagulation.

1. *Determination of Gastrin*. 10 g/L of ethylenediamine tetraacetic acid (EDTA) at a dose of 30 *μ*L and trypsin inhibitor at a dose of 20 *μ*L were injected into the test tube. After the sufficient mixing, the mixture was in static condition for 4 h and then centrifuged at 3000 r/min for 15 min to separate the plasma, using an ultralow temperature refrigerator at −80°C to freeze and store. The freeze-thaw was taken out during the determination, using the same test kits (manufacturer: Shanghai Shuangying Biotechnology Co., Ltd.) to determine
2. *Determination of IGF-1 and GHBP*. After the blood was fully solidified, it was centrifuged at 3000 r/min for 5 min by a centrifuge to obtain the serum using a refrigerator at -20°C to store. After all samples were collected, the IGF-1 was determined by chemiluminescence immunoassay, and the GHBP was measured by enzyme-linked immunosorbent assay. All operations were carried out according to the relevant instructions of the kits (manufacturer: Beijing Kerui Mei Technology Co., Ltd.)

#### 2.3.2. Detection of Growth and Development Indices

1. *Measurement of Height and Weight*. The subjects kept standing in attention position on the floor of the height and weight measuring instrument, with the naturally upright body. The heels, shoulders, and sacral tail were in contact with the column, with the head in frontal position, while children kept looking straight ahead. The height and weight were measured three times in order to obtain the average value
2. *Measurement of BMDZ-Scores*. The BMD *Z*-scores were measured by an ultrasound bone densitometer (manufacturer: Xuzhou Pinyuan Electronic Technology Co., Ltd.; model: BMD-A1). 1/3 of the left distal tibia as the measurement site was taken three times to obtain the average value. The determination criteria were as follows. If the measurement value was −2.0 ≤ *Z* < −1.0, the BMD was mild deficiency. If the measurement value was −3.0 ≤ *Z* < −2.0, the BMD was moderate deficiency. If the measurement value was *Z* < −3.0, the BMD was severe deficiency

### 2.4. Intervention Methods

Children in the study group were treated with the recombinant human growth hormone (manufacturer: Shenzhen Kexing Biotech Co., Ltd.; NMPA approval no.: S20063087; specification: 0.65 mg/piece) by the subcutaneous injection at a dose of 0.05 mg/time every day and lysine hydrochloride tablets (manufacturer: Anqiu Lu'an Pharmaceutical Co., Ltd.; NMPA approval No.: H37023859; specification: 0.2 g) by oral administration at a dose of 200 mg/time every day.

### 2.5. Observation Indices

The growth and development indices (height, weight and BMD *Z*-scores) and the levels of gastrin, serum IGF-1, and GHBP were compared between the two groups, and the correlation between the levels of gastrin, serum IGF-1, and GHBP and the growth and development indices was analyzed.

The levels of gastrin, serum IGF-1, and GHBP before and after treatment in children with SS were compared.

### 2.6. Statistical Methods

The data included in this study were processed by the professional statistical software SPSS26.0, and the pictures were drawn by the GraphPad Prism 7 (GraphPad Software, San Diego, USA). The enumeration data and measurement data were tested by *x*^2^ and *t* test, indicated by (*n* (%)) and Mean ± SD. The Pearson correlation coefficient method was used to analyze the correlation between the levels of gastrin, serum IGF-1, and GHBP and growth and development indices in children. When *P* < 0.05, the differences were considered to be statistically significant.

## 3. Results

### 3.1. Comparison of Growth and Development Indices

Compared with the study group, the height, weight, and BMD *Z*-scores of children in the control group were obviously higher (*P* < 0.001), see details in Table 1.

### 3.2. Comparison of Levels of Gastrin, Serum IGF-1, and GHBP

**Figure 1:**
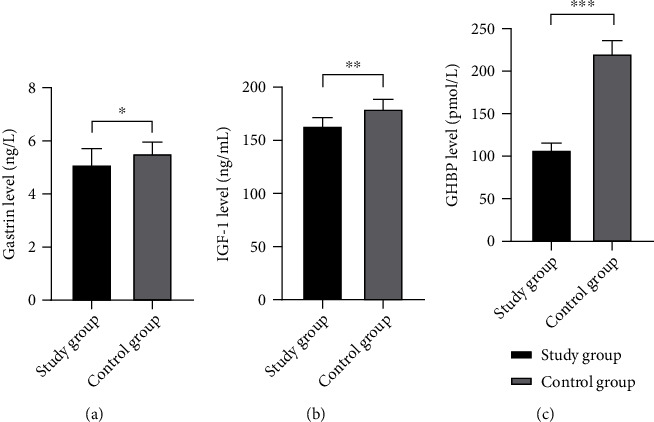
Comparison of levels of gastrin, serum IGF-1, and GHBP in children between the two groups (Mean ± SD). Notes. (a) was the comparison of the gastrin levels between the two groups. The transverse axis represented the study group and the control group, and the vertical axis represented the gastrin level (ng/mL). The gastrin levels in the study group and the control group were (5.11 ± 0.60) ng/mL and (5.53 ± 0.43) ng/mL, respectively. ^∗^ represented a significant difference in the gastrin levels between the two groups in children (*t* = 3.277, *P* < 0.05). (b) was the comparison of the IGF-1 levels between the two groups. The transverse axis represented the study group and the control group, and the vertical axis represented the IGF-1 level (ng/mL). The IGF-1 levels in the study group and the control group were (163.80 ± 7.64) ng/mL and (179.73 ± 8.81) ng/mL, respectively. ^∗∗^ represented a significant difference in the IGF-1 levels between the two groups in children (*t* = 8.182, *P* < 0.001). (c) was the comparison of the GHBP levels between the two groups. The transverse axis represented the study group and the control group, and the vertical axis represented the GHBP level (pmol/L). The GHBP levels in the study group and the control group were (108.92 ± 4.43) pmol/L and (222.07 ± 15.36) pmol/L, respectively. ^∗∗∗^ represented a significant difference in the GHBP levels between the two groups in children (*t* = 45.289, *P* < 0.001).

The results showed that the levels of gastrin, serum IGF-1, and GHBP of children in the study group were markedly lower than those in the control group (*P* < 0.05), see details in Figure 1.

### 3.3. Correlation between the Levels of Gastrin, Serum IGF-1, and GHBP and Growth and Development Indices

The Pearson correlation analysis showed that the gastrin, serum IGF-1, and GHBP of children were positively correlated with growth and development indices (*P* < 0.001), see details in Table 2.

### 3.4. Comparison of Levels of Gastrin, Serum IGF-1, and GHBP in Children before and after Treatment

**Figure 2:**
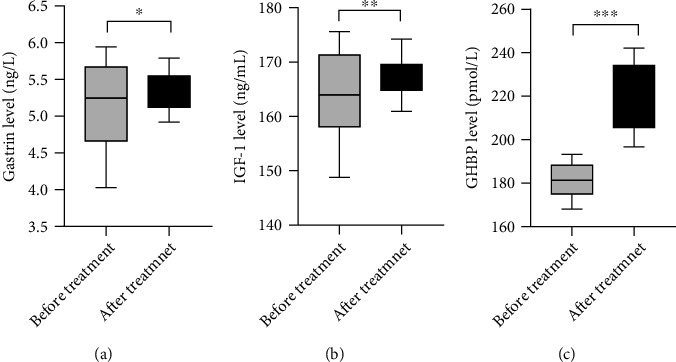
Comparison of levels of gastrin, serum IGF-1, and GHBP in children before and after treatment (Mean ± SD). Notes. (a) showed the comparison of gastrin levels in children before and after treatment. The transverse axis represented before and after treatment, and the vertical axis represented the gastrin level (ng/mL). The gastrin levels in children before and after treatment were (5.11 ± 0.60) ng/mL and (5.34 ± 0.27) ng/mL, respectively. ^∗^ represented a significant difference in the gastrin levels of children before and after treatment (*t* = 2.265, *P* < 0.05). (b) showed the comparison of IGF-1 levels in children before and after treatment. The transverse axis represented before and after treatment, and the vertical axis represented the IGF-1 level (ng/mL). The IGF-1 levels in children before and after treatment were (163.80 ± 7.64) ng/mL and (167.18 ± 3.67) ng/mL, respectively. ^∗∗^ represented a significant difference in the IGF-1 levels of children before and after treatment (*t* = 2.584, *P* < 0.05). (c) showed the comparison of GHBP levels in children before and after treatment. The transverse axis represented before treatment and after treatment, and the vertical axis represented the GHBP level (pmol/L). The GHBP levels in children before and after treatment were (181.53 ± 7.45) pmol/L and (220.92 ± 15.31) pmol/L, respectively. ^∗∗∗^ represented a significant difference in the GHBP levels of children before and after treatment (*t* = 14.993, *P* < 0.001).

The study results showed that the levels of gastrin, serum IGF-1, and GHBP were significantly improved after treatment in children (*P* < 0.05), see details in Figure 2.

## 4. Discussion

There are many factors affecting the growth and development of children, with a relatively complex mechanism, and SS has been discussed by many researchers [10, 11]. Some studies have shown that the function abnormality of hypothalamus-pituitary and its IGF axis is the main reason affecting the growth and development of children [12]. IGF-1, a peptide and protein whose structure is similar to the insulin factor, is secreted by kidney, liver, and spleen cells. The growth hormone (GH)/IGF-1 axis, as a study hotspot in recent years, can regulate the bone balance in human body and determine the growth length, maturity of bone, and the obtained maximum bone mass before puberty [13], and its main manifestation is to maintain the bone mass at adult stage. IGF-1 can directly promote the cell differentiation and proliferation as a target cell, and GH can stimulate the IGF-1 produced by peripheral tissues especially liver. In addition, IGF-1 promotes the growth mediated by GH [14] and induces the occurrence of chondrocyte mitosis, both of which participate in the growth and development of children. Animal experiments have found [15, 16] that GHBP, a specific binding protein of GH in plasma, can bind to the GH, which can reduce the fluctuation of GH level caused by the pulsatile secretion of GH in hypophysis and prolong the half-life of GH, thus regulating the GH. GHBP in human is generated by the protein cleavage mechanism of growth hormone receptor (GHR), which can be used to reflect the status of GHR in the body. Therefore, in a sense, GHBP is able to provide a theoretical basis for the clinical treatment of children with SS [17, 18]. In addition, the related studies of GHBP have achieved remarkable results in other areas. Some scholars [19] have proposed that serum GHBP can be used as an important biological marker for screening of Down syndrome in the early pregnant stage, and its level is also positively correlated with the risk of breast cancer in women [20].

Gastrin is a peptide hormone released into the blood by the secretion of gastric fundus, with the element of 28 amino acids [21]. After acylation, it is able to combine with the growth hormone secretagogue receptor to across the blood-brain barrier, which is the only known hormone in the blood to stimulate appetite, with an unclear mechanism on promoting the GH secretion. Current studies have shown that [22] gastrin can increase the circulating level of GH by at least two pathways. A pathway is to increase the cycle of phosphoric acid and activate the protein kinase C by activating the signaling pathway of phospholipase C, which can cause the increase of intracellular Ca^2+^ level and the release of diacylglycerol, thereby increasing the secretion of growth hormone-releasing hormone. The second pathway is that gastrin directly increases the GH secreted by pituitary cells, and the specific regulation pathway remains to be further studied.

In recent years, the real world study based on daily practice data in clinical care has been paid more and more attention, and big data application in medical field has been listed as the key development direction in China [23, 24]. In this study, the laboratory tests were used to analyze the differences of growth and development indices, gastrin, serum IGF-1, and GHBP between children with SS and healthy children by big data, hoping to determine the correlation between the levels of gastrin, serum IGF-1, and GHBP and growth and development in children with SS by comparing the clinical data. The result showed that the levels of gastrin, serum IGF-1, and GHBP of children in the study group were significantly lower than those in the control group (*P* < 0.05), and the result enabled people to have a new understanding of SS, which was obviously better than that of Motta Felipe et al. [25]. The Pearson correlation analysis showed that there were significant correlations between gastrin, serum IGF-1, and GHBP and growth and development indices in children (*P* < 0.001). The results of the proposed method in this study were compared with those of the traditional methods, which exceeded the previous reports, indicating that the experiment had a higher clinical value. The contributions of this study were as below. The relationship between serological indicators and growth and development indices in children with SS was analyzed to more deeply understand the pathogenesis of SS, which was a major progress and development in clinic undoubtedly, with extensive guiding significance in the treatment of SS and as the development direction of future medicine. The advantages of this study were that the relationship between serological indicators and growth and development indices of children in the two groups was detected, and the relation of test variables was explored using the Pearson correlation analysis, which was innovative undoubtedly. The limitation of this study was as follows. Only 42 cases were included in this study, with a small sample size, so that there must be a deviation in sample selection, and the large-sample and multicenter studies are needed in the subsequent studies.

## Figures and Tables

**Table 1:** Comparison of growth and development indices in children between the two groups (Mean ± SD).

Groups	*n*	Height (cm)	Weight (kg)	BMD *Z*-scores
Study group	42	103.36 ± 6.02	18.40 ± 2.89	−1.18 ± 0.54
Control group	30	114.67 ± 7.94	26.05 ± 6.60	0.44 ± 0.07
*t*		6.876	7.075	7.446
*P*		<0.001	<0.001	<0.001

**Table 2:** Correlation between the levels of gastrin, serum IGF-1, and GHBP and growth and development indices.

Observation indices	Gastrin		Serum IGF-1		Serum GHBP	
	*r* values	*P* values	*r* values	*P* values	*r* values	*P* values
Height	0.822	<0.001	0.673	<0.001	0.758	<0.001
Weight	0.653	<0.001	0.520	<0.001	0.706	<0.001
BMD *Z*-scores	0.621	<0.001	0.544	<0.001	0.686	<0.001

## Data Availability

Data to support the findings of this study is available on reasonable request from the corresponding author.
